# Brassinin Promotes the Degradation of Tie2 and FGFR1 in Endothelial Cells and Inhibits Triple-Negative Breast Cancer Angiogenesis

**DOI:** 10.3390/cancers14143540

**Published:** 2022-07-21

**Authors:** Yuan Gu, Vivien Becker, Moqin Qiu, Tianci Tang, Emmanuel Ampofo, Michael D. Menger, Matthias W. Laschke

**Affiliations:** 1 Institute for Clinical and Experimental Surgery, Saarland University, 66421 Homburg, Germany; vivien.becker@uks.eu (V.B.); qiumoqin@stu.gxmu.edu.cn (M.Q.); tianci.tang@uks.eu (T.T.); emmanuel.ampofo@uks.eu (E.A.); michael.menger@uks.eu (M.D.M.); matthias.laschke@uks.eu (M.W.L.); 2 Department of Respiratory Oncology, Guangxi Medical University Cancer Hospital, Nanning 530021, China

**Keywords:** angiogenesis, brassinin, dorsal skinfold chamber model, endothelial cell, FGFR1, microvessel, phytochemical, Tie2, triple-negative breast cancer

## Abstract

**Simple Summary:**

Brassinin is a natural compound enriched in several commonly consumed vegetables, such as broccoli and cabbages. It shows potent anti-cancer activity against several cancers. However, its effects on triple-negative breast cancer (TNBC), an aggressive subtype with limited treatment options, remain elusive so far. Therefore, we investigated the effects of brassinin on TNBC angiogenesis and growth. Our results demonstrate that brassinin inhibits TNBC growth preferentially through inhibiting the angiogenic activity of endothelial cells (ECs). Additional in-vitro analyses revealed that this effect may be mediated by brassinin-stimulated degradation of two pivotal angiogenesis-related receptors in ECs: Tie2 and fibroblast growth factor receptor 1. These findings provide novel insights into the cellular and molecular mechanisms underlying the anti-cancer activity of brassinin and indicate that this phytochemical may be a promising lead compound or drug candidate for TNBC treatment.

**Abstract:**

Brassinin, a phytoalexin derived from cruciferous vegetables, has been reported to exhibit anti-cancer activity in multiple cancer types. However, its effects on triple-negative breast cancer (TNBC) development and the underlying mechanisms have not been elucidated so far. In this study, we demonstrated in vitro that brassinin preferentially reduces the viability of endothelial cells (ECs) when compared to other cell types of the tumor microenvironment, including TNBC cells, pericytes, and fibroblasts. Moreover, brassinin at non-cytotoxic doses significantly suppressed the proliferation, migration, tube formation, and spheroid sprouting of ECs. It also efficiently inhibited angiogenesis in an ex-vivo aortic ring assay and an in-vivo Matrigel plug assay. Daily intraperitoneal injection of brassinin significantly reduced tumor size, microvessel density, as well as the perfusion of tumor microvessels in a dorsal skinfold chamber model of TNBC. Mechanistic analyses showed that brassinin selectively stimulates the degradation of Tie2 and fibroblast growth factor receptor 1 in ECs, leading to the down-regulation of the AKT and extracellular signal-regulated kinase pathways. These findings demonstrate a preferential and potent anti-angiogenic activity of brassinin, which may be the main mechanism of its anti-tumor action. Accordingly, this phytochemical represents a promising candidate for the future anti-angiogenic treatment of TNBC.

## 1. Introduction

Phytochemicals represent a rich source of effective, safe, and cheap compounds that have great potential in cancer prevention and treatment. Such a compound is brassinin, a phytoalexin isolated from cruciferous vegetables, including cabbage, broccoli, and mustard greens [1]. Previous in-vitro studies demonstrated anti-proliferative and cytotoxic effects of brassinin on human colorectal [2,3], liver [4], and lung cancer cells [5]. In addition, brassinin has been shown to enhance the anti-cancer effects of paclitaxel in a A549 lung cancer xenograft mouse model [5] and an autochthonous mouse model of mammary gland tumors [6]. However, the exact cellular and molecular mechanisms underlying the anti-cancer activity of brassinin, especially its anti-angiogenic activity, have not been clarified yet. 

Inhibition of angiogenesis, i.e., the outgrowth of new blood vessels from pre-existing ones, represents a promising anti-cancer strategy. In fact, when a tumor exceeds a few millimeters in diameter, hypoxia and nutrient deprivation drive tumor cells to secrete various pro-angiogenic factors, including vascular endothelial growth factor (VEGF) and fibroblast growth factor (FGF) [7]. These factors, in turn, bind to their receptors on the surface of endothelial cells (ECs), causing the activation of pivotal signaling pathways associated with angiogenesis, such as phosphatidylinositol 3-kinase/protein kinase B (AKT)/mammalian target of rapamycin (mTOR), rapidly accelerated fibrosarcoma (Raf)/mitogen-activated protein kinase (MAPK) and extracellular signal-regulated kinase (ERK) kinase (MEK)/ERK [8,9]. As a consequence, ECs are activated to proliferate, migrate, and reorganize into new capillaries. The newly formed blood vessels not only provide oxygen and nutrients for tumor growth but also an escape route for tumor metastasis [7]. 

To date, several neutralizing antibodies against VEGFs and their receptors (VEGFRs) as well as tyrosine kinase inhibitors have been approved by the United States Food and Drug Administration for the anti-angiogenic treatment of solid tumors [10]. However, the clinical benefits of these therapies are quite limited due to the onset of intrinsic tumor resistance [10,11]. This resistance arises either from overexpression of target proteins or redundancy in alternative angiogenic pathways [10]. Thus, the development of safe and effective anti-angiogenic drugs is urgently needed.

For these reasons, we studied whether brassinin exerts anti-angiogenic effects. For this purpose, we first analyzed the selectivity of brassinin against ECs in comparison to other cell types of the tumor microenvironment, including breast cancer cells, pericytes, and fibroblasts, by means of cell viability assays. To unravel the effects of brassinin on the angiogenic activities of ECs, we further performed a panel of in-vitro and in-vivo angiogenesis assays. In addition, we analyzed the action of brassinin on the growth and vascularization of triple-negative breast cancer (TNBC) in a mouse dorsal skinfold chamber model. TNBC is the most aggressive breast cancer subtype with a very poor prognosis and limited treatment options [12,13]. Finally, we investigated the molecular mechanisms underlying the anti-angiogenic effects of brassinin. 

## 2. Materials and Methods

### 2.1. Study Design

In this study, the sample size chosen for each assay was based on previous publications [14,15,16,17]. For in-vitro assays, at least three independent experiments with at least three biological replicates (i.e., independent cell cultures) were performed. For mouse models, 6–7 mice were included in each group. All the analyses were carried out by the investigators who were blinded to the group allocation. No samples or animals were excluded from the analysis.

### 2.2. Chemicals 

Brassinin, cycloheximide, and MG132 were purchased from Santa Cruz Biotechnology (Heidelberg, Germany). Chloroquine diphosphate salt was purchased from Sigma-Aldrich (Taufkirchen, Germany).

### 2.3. Cell Culture 

Human umbilical vein endothelial cells (HUVECs) and human dermal microvascular endothelial cells (HDMECs) were purchased from PromoCell (Heidelberg, Germany) and cultured in SupplementMix-supplemented Endothelial Cell Growth Medium (EGM; PromoCell) and EGM-MV (PromoCell), respectively. Human pericytes from placenta (hPC-PLs; PromoCell) were cultured in Pericyte Growth Medium 2 (PromoCell) with SupplementMix (PromoCell). Normal human dermal fibroblasts (NHDFs; a kind gift from Dr. Wolfgang Metzger at Saarland University) were cultured in Dulbecco’s modified Eagle’s medium (PAA, Cölbe, Germany) supplemented with 10% fetal calf serum (FCS), 100 U/mL penicillin (PAA) and 0.1 mg/mL streptomycin (PAA). The murine TNBC cell line 4T1 (ATCC, Wesel, Germany), the human TNBC cell line MDA-MB-231 (ATCC), and the human non-TNBC cell line MCF-7 (ATCC) were cultured in RPMI 1640 medium with 10% FCS, 100 U/mL penicillin, and 0.1 mg/mL streptomycin. All cells were maintained in a humidified incubator containing 5% CO_2_ at 37 °C. 

### 2.4. Water-Soluble Tetrazolium (WST)-1 Assay

To assess cell viability, WST-1 assays (Roche Diagnostics, Mannheim, Germany) were performed according to the manufacturer’s instructions. Briefly, 2–3 × 10³ cells were seeded into each well of 96-well plates. After overnight incubation, the cells were treated with vehicle (0.1% *v*/*v* DMSO) or serial dilutions of brassinin. After 48 h of treatment, 10 µL WST-1 reagent was added into each well and the plates were placed in a cell incubator for 30 min. The absorbance of each well was measured at 450 nm by a PHOmo microplate reader (anthos Mikrosysteme GmbH, Krefeld, Germany). 

### 2.5. Lactate Dehydrogenase (LDH) Assay

Following the manufacturer’s instructions (Roche Diagnostics), HUVECs seeded in 96-well plates (4 × 10^3^ cells per well) were exposed to brassinin at different concentrations for 24 h. Subsequently, 100 µL LDH reaction mix was added to each well followed by 10 min of incubation at room temperature. After the addition of stop solution (50 µL per well), the plates were measured at 492 nm in a microplate photometer (PHOmo). A wavelength of 620 nm served as reference.

### 2.6. Bromodeoxyuridine (BrdU) Incorporation Assay

We performed a BrdU incorporation assay to assess EC proliferation, as described previously with minor modifications [14]. Briefly, 2.5 × 10^5^ HUVECs were seeded into each well of 6-well plates. After overnight incubation, the cells were treated with different concentrations of brassinin for 6 h. Subsequently, BrdU was added into the cell medium to a final concentration of 10 µM. After an additional 18 h of incubation, the cells were fixed in 70% ethanol and denatured in 2 M hydrochloric acid containing 0.5% Triton X-100. Thereafter, the cells were stained with an anti-BrdU antibody labeled with fluorescein isothiocyanate (1:100; Thermo Fisher Scientific, Karlsruhe, Germany). Sample detection was performed using a FACScan flow cytometer (BD Biosciences, Heidelberg, Germany) and BrdU-positive cells were counted and expressed as a percentage of the total cell number.

### 2.7. Transwell Migration Assay

EC motility was analyzed by means of transwell migration assays. Briefly, HUVECs maintained in culture dishes were treated for 24 h with brassinin at different concentrations. Subsequently, 500 µL endothelial basal medium containing 1.5 × 10^5^ treated HUVECs was added into an insert of 24-transwell plates (pore size: 8 µm; Corning; Merck KGaA, Darmstadt, Germany), while 750 µL endothelial basal medium containing 1% FCS was added to the lower well. After incubating the plates for 5 h, the unmigrated cells were removed with cotton swaps, while the migrated cells were dyed with Diff-Quick (LT-SYS Diagnostika, Berlin, Germany). Cell migration was quantified by counting the number of migrated ECs in 20 fields at 200× magnification under a BZ-8000 microscope (Keyence, Osaka, Japan) and expressed as a percentage of the vehicle-treated control group.

### 2.8. Tube Formation Assay

To check the tube-forming activity of ECs, 1.7 × 10^4^ HUVECs were suspended in EGM containing different concentrations of brassinin and seeded into each well of 96-well plates that were pre-coated with 50 µL Matrigel (Corning; Merck KGaA). After 24 h of incubation, the newly formed tubes were photographed under a phase-contrast microscope (BZ-8000; Keyence). Tube formation was quantified by analyzing the quantity of meshes using the angiogenesis analyzer plugin of ImageJ (U.S. National Institutes of Health, Bethesda, MD, USA) and expressed as a percentage of the vehicle-treated control group.

### 2.9. Spheroid Sprouting Assay

As we previously described [15], 500 HUVECs in culture medium containing 20% methylcellulose (Thermo Fisher Scientific) were seeded in 96-well round bottom non-adherent plates and cultured for 24 h. Afterwards, spheroids were pelleted and resuspended in a polymerization solution, which was composed of 2 volumes basal medium containing 0.5% methylcellulose and 20% FCS and 1 volume collagen solution. The collagen solution was prepared with rat acidic collagen extract (Serva, Heidelberg, Germany), 10× Medium 199 (Sigma-Aldrich), and 0.2 M NaOH at a ratio of 8:1:1. Subsequently, a spheroid mixture (~50 spheroids) was placed into each well of a pre-warmed 24-well plate. After incubation for 45 min, the spheroids were exposed to different concentrations of brassinin for 24 h and then photographed by a phase-contrast microscope (DFC450C; Leica Microsystems, Wetzlar, Germany). Spheroid sprouting was quantified by measuring the cumulative sprout length, i.e., the overall length of all sprouts growing out of each spheroid, by utilizing the Leica LAS V4.8 software and expressed as a percentage of the vehicle-treated control group.

### 2.10. Aortic Ring Assay

Aortic rings processed from male 8-week-old Balb/c mice were embedded in 50 µL Matrigel (Corning; Merck KGaA) in each well of 96-well plates (one ring per well). After incubating the plates for 15 min, Dulbecco’s modified Eagle’s medium containing 10% FCS and different concentrations of brassinin was added gently onto the Matrigel. After culturing for 6 days, the aortic rings were photographed using a phase-contrast microscope (BZ-8000; Keyence). Aortic sprouting was quantified by measuring the area of the outer aortic vessel sprouting and expressed as a percentage of the vehicle-treated control group.

### 2.11. Animal Models

The in-vivo effects of brassinin on angiogenesis were evaluated in a Matrigel plug assay as previously described with minor modifications [16]. Briefly, 300 µL growth factor-reduced Matrigel (~7 mg/mL; Corning; Merck KGaA) containing 1 µg/mL VEGF (R&D Systems, Wiesbaden, Germany), 1 µg/mL basic FGF (R&D Systems), 50 IU/mL heparin (B. Braun, Melsungen, Germany), and 100 µM brassinin or DMSO (0.1% *v/v*; vehicle) was subcutaneously injected into 8–10-week-old Balb/c mice (*n* = 6 per group). After 7 days, the Matrigel plugs were harvested for immunohistochemical analyses.

To investigate the anti-cancer effects of brassinin on TNBC, a mouse dorsal skinfold chamber model was performed as described previously in detail [17]. Briefly, 4T1 spheroids were firstly prepared by seeding 5 × 10^4^ cells into each well of 96-well plates that were coated with 1% agarose, followed by 3 days of incubation. One day after cell seeding, dorsal skinfold chambers were implanted into female 12–15-week-old Balb/c mice. After another 2 days, one 4T1 spheroid stained with Hoechst 33342 was transplanted into each chamber. The mice were then randomly assigned to two groups (*n* = 7 per group) and injected intraperitoneally with 180 mg/kg brassinin or vehicle (DMSO:corn oil = 1:4; 50 µL/25 g mouse) once a day for 14 consecutive days. Tumor vascularization and growth were monitored by intravital fluorescence microscopy every 3–4 days after spheroid transplantation. The microscopic images were recorded by a FK6990 CCD video camera (Pieper, Schwerte, Germany) and analyzed using CapImage (Zeintl, Heidelberg, Germany) to quantify the tumor size, the functional microvessel density in each tumor (i.e., the total length of red blood cell-perfused tumor microvessels divided by the observation area), as well as the diameter (D; μm), the centerline red blood cell velocity (V; μm/s), and the volumetric blood flow (pL/s) of tumor microvessels. The volumetric blood flow was calculated as π × (D/2)² × V/1.3 [18,19]. At the end of the experiment, i.e., on day 14, the dorsal skinfold chamber preparations were excised and the tumor tissue was processed for further histological and immunohistochemical analyses.

### 2.12. Histology and Immunohistochemistry

Excised dorsal skinfold chamber preparations or Matrigel plugs were fixed in formalin, embedded in paraffin, and cut into 3-μm sections. To analyze the size of tumors, the vertical cross-sections of dorsal skinfold chamber preparations that presented the largest area of each tumor were stained with hematoxylin and eosin. Then, these sections were photographed under a BZ-8000 microscope (Keyence) and the tumor size was measured using the Keyence image analysis software. To detect microvessels in dorsal skinfold chamber tumors and Matrigel plugs, the sections were subsequently incubated with a rabbit anti-mouse CD31 antibody (1:100; Abcam, Cambridge, UK; ab182981), a goat anti-rabbit secondary antibody labeled with Alexa Fluor 555 (1:100; Thermo Fisher Scientific; A27039) and Hoechst 33342 (2 µg/mL; Sigma-Aldrich). Eight fields of each section were photographed under a fluorescence microscope (BX60; Olympus, Hamburg, Germany) at 200× magnification and analyzed to determine the microvessel density by counting the number of CD31-positive vessels in each field. To assess tumor cell proliferation and apoptosis, the sections were incubated with a rabbit anti-mouse Ki67 antibody (1:500; Cell Signaling Technology, Frankfurt, Germany; #12202) and a rabbit anti-mouse cleaved caspase-3 antibody (1:100; Cell Signaling Technology; #9661), respectively, followed by the corresponding biotin-conjugated secondary antibody (Abcam; ab64256) and horseradish peroxidase (HRP)-conjugated streptavidin (Abcam). Then, the sections were incubated with the peroxidase substrate 3-amino-9-ethylcarbazole (Abcam) and counterstained with Mayer’s hemalum solution (Merck KGaA). Twelve fields of each section were photographed under the BX-60 microscope (Olympus) at 400× magnification and analyzed to quantify the percentage of Ki67-positive and cleaved caspase-3-positive tumor cells.

### 2.13. Western Blotting

As previously described in detail [20], whole cell lysates were prepared in RIPA lysis buffer (Thermo Fisher Scientific) containing phenylmethylsulfonyl fluoride and Protease Inhibitor Cocktail (Sigma-Aldrich). After centrifugation, the supernatant of the cell lysate was processed for the determination of protein concentration using a Pierce BCA Protein Assay Kit (Thermo Fisher Scientific). Subsequently, equal amounts of protein (15 µg) were separated via 8% sodium dodecyl sulphate-polyacrylamide gel electrophoresis and then electrotransferred onto polyvinylidene difluoride membranes (BioRad, Munich, Germany). Then, the membranes were blocked with milk (BioRad) or bovine serum albumin (Thermo Fisher Scientific) and incubated with a rabbit monoclonal anti-VEGFR2 antibody (1:300; Cell Signaling Technology; #9698), a rabbit polyclonal anti-VEGFR1 antibody (1:300; Abcam; ab32152), a rabbit monoclonal anti-FGF receptor (FGFR) 1 antibody (1:300; Cell Signaling Technology; #9740), a rabbit monoclonal anti-Tie2 antibody (1:300; Cell Signaling Technology; #7403), a rabbit monoclonal anti-phosphorylated AKT (p-AKT) antibody (1:300; Cell Signaling Technology; #4060), a rabbit anti-AKT antibody (1:500; Cell Signaling Technology; #4685), a mouse anti-phosphorylated-ERK (p-ERK) antibody (1:500; Abcam; ab50011), a rabbit anti-ERK antibody (1:500; Abcam; ab115799) or a mouse anti-β-actin antibody (1:3000, Sigma-Aldrich; A5441), followed by an anti-rabbit (1:1000; R&D Systems; HAF008) or anti-mouse (1:1000; R&D Systems; HAF007) secondary antibody conjugated to HRP. Signals of protein bands were visualized using an enhanced chemiluminescence kit (GE Healthcare, Freiburg, Germany) and images were acquired under a ChemoCam Imager (Intas, Göttingen, Germany). Expression levels of proteins were quantified by analyzing the intensity of each protein band using the ImageJ software, corrected by β-actin or its unphosphorylated form and expressed as a percentage of the vehicle-treated control group.

### 2.14. Quantitative Real-Time Polymerase Chain Reaction (PCR)

Total RNA was extracted from HUVECs that were treated with vehicle or brassinin using a RNeasy Mini kit (Qiagen, Hilden, Germany). Then, equal amounts of total RNA (1 μg) were reversely transcribed using a QuantiNova Reverse Transcription Kit (Qiagen). Quantitative real-time PCR was conducted using a QuantiNova SYBR green PCR kit (Qiagen) on a BioRad MiniOpticon Real-Time PCR System. The messenger RNA (mRNA) levels of genes were calculated using the 2^−ΔΔCt^ method with GAPDH as an endogenous control and expressed as a percentage of vehicle-treated control group. The specific primer sequences are listed as follows: 5′-TTAGCCAGCTTAGTTCTCTGTGG-3′ (forward) and 5′-AGCATCAGATACAAGAGGTAGGG-3′ (reverse) for human Tie2; 5′-GGCTACAAGGTCCGTTATGCC-3′ (forward) and 5′-GATGCTGCCGTACTCATTCTC-3′ (reverse) for human FGFR1; 5′-ATGGGTGTGAACCATGAGAAGTA-3′ (forward) and 5′-GGCAGTGATGGCATGGAC-3′ (reverse) for human GAPDH.

### 2.15. Statistics 

Statistical analysis was performed by means of GraphPad Prism 9.1.0 (GraphPad, La Jolla, CA, USA). Comparisons between two groups were made with the unpaired two-tailed t test, while comparisons among multiple groups were made with One-Way ANOVA followed by Tukey’s multiple comparisons test. All data are expressed as means ± SEM. Statistical significance was accepted for *p* < 0.05 (* *p* < 0.05; ** *p* < 0.01; *** *p* < 0.001).

## 3. Results

### 3.1. Brassinin Preferentially Targets ECs

**Figure 1:**
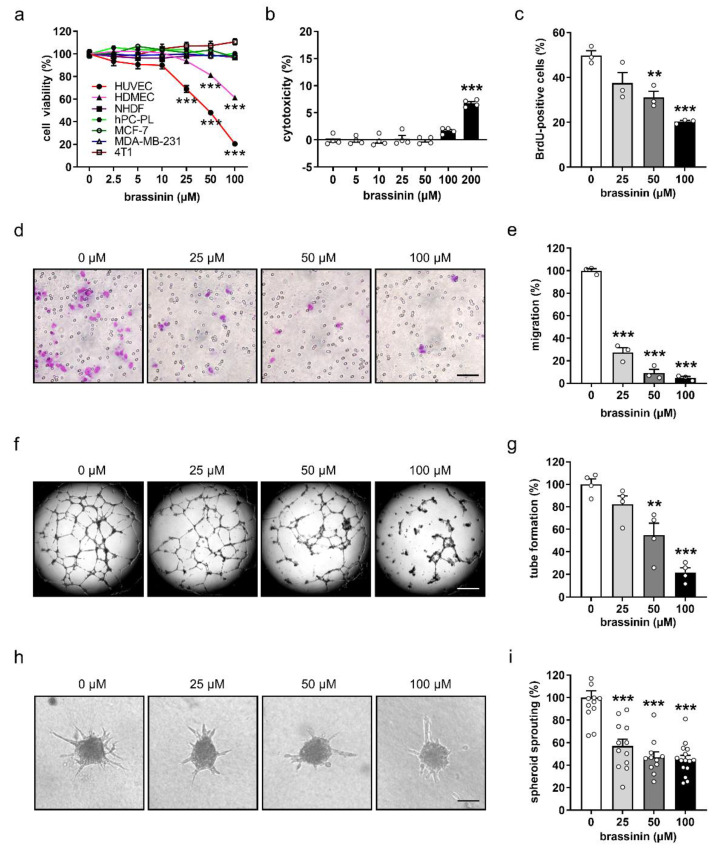
Brassinin preferentially targets ECs and suppresses their angiogenic activity. (**a**) Cell viability (% of 0 µM) of HUVECs, HDMECs, hPC-PLs, and NHDFs, as well as MCF-7, MDA-MB-231, and 4T1 cells after 48 h exposure to a serial dilution of brassinin (*n* = 4). (**b**) Cytotoxicity (% of the total cell death) of brassinin on HUVECs after 24 h exposure to a serial dilution of the compound (*n* = 4). (**c**) BrdU-positive HUVECs (% of the total cell number) that were treated for 24 h with 0, 25, 50, and 100 µM brassinin (*n* = 3). (**d**) Images of migrated HUVECs that were treated for 24 h with 0, 25, 50, and 100 µM brassinin. Scale bar: 70 µm. (**e**) Migration (% of 0 µM) of treated HUVECs shown in (**d**) was quantified (*n* = 4). (**f**) Images of tubes formed by HUVECs. The cells were treated for 18 h with 0, 25, 50, and 100 µM brassinin. Scale bar: 700 µm. (**g**) Tube formation (% of 0 µM) of treated HUVECs shown in (**f**) was determined (*n* = 4). (**h**) Images of HUVEC spheroids that were treated for 24 h with 0, 25, 50, and 100 µM brassinin. Scale bar: 95 µm. (**i**) Sprouting (% of 0 µM) of treated HUVEC spheroids shown in (**h**) was quantified (*n* = 11–15). Means ± SEM. ** *p* < 0.01, *** *p* < 0.001 vs. 0 µM.

The tumor microenvironment plays an essential role in promoting tumor angiogenesis, growth, metastasis, and immune evasion [21]. It is a highly heterogeneous milieu composed of diverse cell types, including malignant tumor cells, ECs, pericytes, and fibroblasts [22]. To unravel the effects of brassinin on these cell types, we assessed the viability of HUVECs, HDMECs, hPC-PLs, and NHDFs, as well as MCF-7, MDA-MB-231, and 4T1 cells by means of WST-1 assays after exposing them for 48 h to different doses of the compound. Of interest, brassinin at doses of 25–100 µM significantly reduced the viability of both types of ECs, i.e., HUVECs and HDMECs (Figure 1a). In contrast, the viability of pericytes, fibroblasts, and breast cancer cells was not affected at all after treatment with the identical doses of brassinin (Figure 1a).

### 3.2. Brassinin Suppresses the Angiogenic Activity of ECs

To investigate the effects of brassinin on the angiogenic activity of ECs, we firstly checked the cytotoxicity of this compound by means of LDH assays. We found that brassinin at concentrations of up to 100 µM exerts no cytotoxicity against HUVECs after 24 h of treatment (Figure 1b). Accordingly, we chose non-cytotoxic concentrations of brassinin, i.e., 25, 50, and 100 µM, for our further angiogenesis assays. In order to analyze the action of brassinin on HUVEC proliferation, the incorporation of BrdU into newly synthesized DNA in proliferating cells was analyzed by flow cytometry. We found that brassinin significantly inhibits EC proliferation in a dose-dependent manner (Figure 1c). Moreover, the effects of brassinin on the migratory activity of ECs were assessed by means of transwell migration assays. Brassinin markedly and dose-dependently reduced the number of migrated HUVECs (Figure 1d,e). We then analyzed the tube-forming capacity of HUVECs in a tube formation assay. Treatment with 50 and 100 µM brassinin caused a 45% and 78% reduction in the number of tube meshes, respectively (Figure 1f,g). In addition, a 3D spheroid sprouting assay revealed that brassinin markedly decreases the cumulative sprout length of HUVEC spheroids in a dose-dependent manner (Figure 1h,i).

### 3.3. Brassinin Inhibits Angiogenesis Ex Vivo and In Vivo

**Figure 2:**
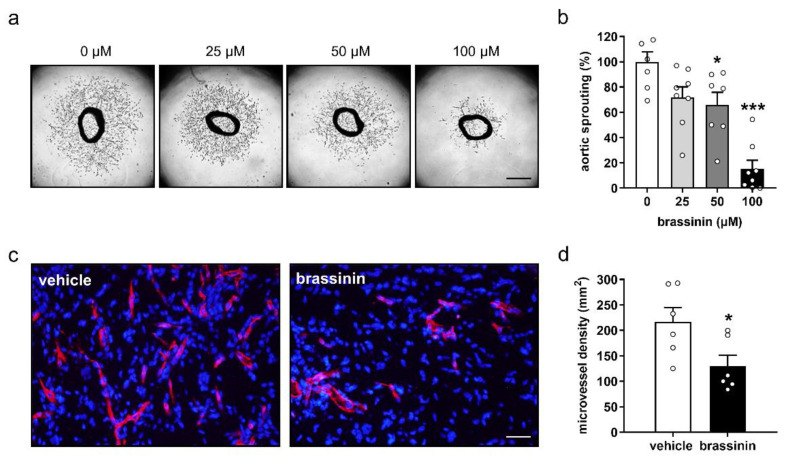
Brassinin inhibits angiogenesis in ex-vivo and in-vivo assays. (**a**) Images of mouse aortic rings that were treated for 6 days with 0, 25, 50, and 100 µM brassinin. Scale bars: 630 µm. (**b**) Sprouting (% of 0 µM) of treated mouse aortic rings shown in (**a**) was quantified (*n* = 7–8). (**c**) Immunohistochemical detection of microvessels (red) in Matrigel plugs containing 100 µM brassinin or DMSO (0.1% *v*/*v*; vehicle). Cell nuclei were stained with Hoechst 33342 (blue). Scale bar: 45 μm. (**d**) Microvessel density (mm^−2^) in Matrigel plugs shown in (**c**) was assessed (*n* = 6). Means ± SEM. * *p* < 0.05, *** *p* < 0.001 vs. 0 µM or vehicle.

To support the in-vitro findings above, we performed an ex-vivo aortic ring assay. This assay is based on the fact that vascular sprouts grow out of the wall of mouse aortic rings when they are cultivated in Matrigel. Of interest, this outgrowth was inhibited in a dose-dependent manner by the treatment with brassinin (Figure 2a). Accordingly, aortic rings exposed to brassinin exhibited a markedly smaller sprout area in comparison to vehicle-treated controls (Figure 2b). 

The effects of brassinin on blood vessel formation were further analyzed in an in-vivo Matrigel plug assay. Matrigel plugs loaded with 100 µM brassinin presented with a 40% reduction of microvessel density when compared to vehicle-treated controls (Figure 2c,d).

### 3.4. Brassinin Inhibits Tumor Growth and Angiogenesis

**Figure 3:**
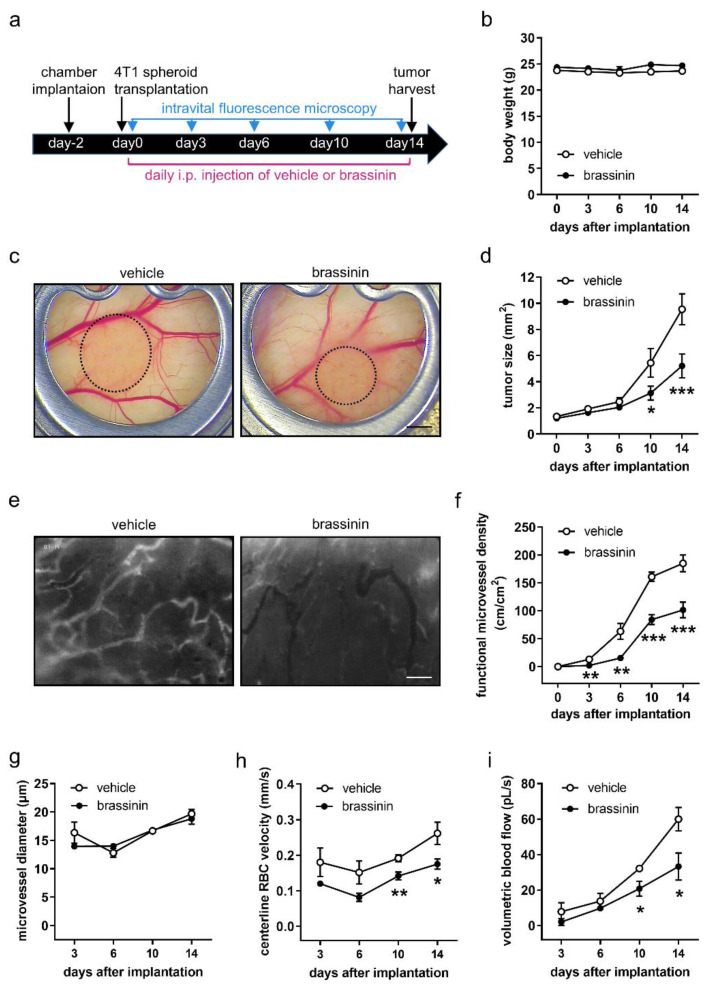
Brassinin inhibits TNBC growth and angiogenesis. (**a**) Timeline of the dorsal skinfold chamber model assessing the effect of brassinin on 4T1 tumor development. (**b**) Body weight (g) of vehicle- and brassinin-treated mice on day 0, 3, 6, 10, and 14 after spheroid transplantation (*n* = 7). (**c**) Stereomicroscopy of 4T1 tumors (outlined with dashed lines) in the dorsal skinfold chamber of a vehicle- and a brassinin-treated mouse on day 14. Scale bar: 1.3 mm. (**d**) Size (mm^2^) of 4T1 tumors on day 0, 3, 6, 10, and 14 after spheroid transplantation was measured (*n* = 7). (**e**) Images of newly formed microvessels within a vehicle- and a brassinin-treated 4T1 tumor on day 14 after spheroid transplantation. Scale bar: 95 µm. (**f**) Functional microvessel density (cm/cm^2^) of 4T1 tumors was quantified (*n* = 7). (**g**–**i**) Microvessel diameter (μm; g), centerline red blood cell velocity (μm/s; h) and volumetric blood flow (pL/s; i) of 4T1 tumors were measured (*n* = 7). Means ± SEM. * *p* < 0.05, ** *p* < 0.01, *** *p* < 0.001 vs. vehicle at each time point.

To investigate the effects of brassinin on TNBC growth and vascularization in vivo, murine 4T1 cell spheroids were transplanted into the dorsal skinfold chamber of syngeneic Balb/c mice. Thereafter, the developing tumors in vehicle- and brassinin-treated mice were repeatedly analyzed by means of intravital fluorescence microscopy (Figure 3a). Importantly, the daily administration of brassinin over 14 days did not affect the body weight of the animals. In both groups, the mice exhibited a stable body weight of ~23–25 g throughout the treatment period (Figure 3b). Moreover, they showed a normal activity as well as feeding and sleeping behavior. However, brassinin administration significantly reduced the size of the developing tumors on day 10 and 14 after tumor spheroid transplantation when compared to controls (Figure 3c,d). Brassinin-treated tumors further exhibited a markedly reduced functional microvessel density between day 3 and 14 (Figure 3e,f; Videos S1 and S2). The analysis of microhemodynamic parameters showed no difference in tumor microvessel diameter between vehicle- and brassinin-treated groups (Figure 3g; Videos S1 and S2), whereas the centerline red blood cell velocity and the volumetric blood flow of tumor microvessels were significantly decreased on day 10 and 14 in brassinin-treated mice (Figure 3h,i; Video S1 and S2).

**Figure 4:**
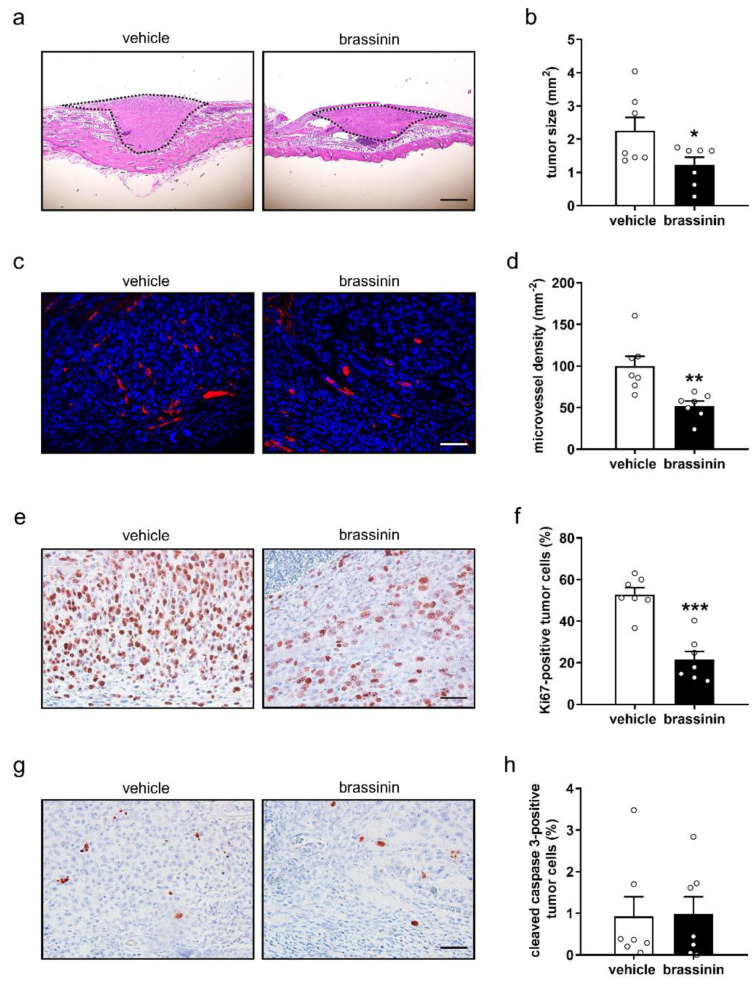
Brassinin reduces TNBC size, vascularization, and tumor cell proliferation. (**a**) Hematoxylin and eosin-stained cross section of 4T1 tumors (outlined with dashed line) within the dorsal skinfold chamber of a vehicle- and a brassinin-treated mouse on day 14 after spheroid transplantation. Scale bars: 595 μm. (**b**) Size (mm^2^) of 4T1 tumors shown in (**a**) was measured (*n* = 7). (**c**) Images of microvessels (red) in 4T1 tumors within dorsal skinfold chambers of a vehicle- and a brassinin-treated mouse on day 14. Cell nuclei were stained with Hoechst 33342 (blue). Scale bar: 55 µm. (**d**) Density (mm^−2^) of tumor microvessels shown in (**c**) was analyzed (*n* = 7). (**e**,**g**) Immunohistochemical detection of Ki67- (**e**) and cleaved caspase-3-positive (g) tumor cells within the dorsal skinfold chambers of a vehicle- and a brassinin-treated mouse on day 14 after spheroid transplantation. Scale bars: 55 µm. (**f**,**h**) Ki67- (**f**) and cleaved caspase-3-positive tumor cells (**h**) (% of the total number of nuclei) were determined (*n* = 7). Means ± SEM. * *p* < 0.05, ** *p* < 0.01, *** *p* < 0.001 vs. vehicle.

Additional histological and immunohistochemical analyses of the tumors on day 14 after spheroid transplantation showed that brassinin-treated tumors presented with a significantly smaller size (Figure 4a,b) and lower microvessel density (Figure 4c,d) when compared to vehicle-treated controls. Moreover, brassinin treatment markedly decreased the number of Ki67-positive proliferating tumor cells (Figure 4e,f), but not the number of cleaved caspase-3-positive apoptotic tumor cells (Figure 4g,h). In both groups, morphological signs of tumor cell necrosis could not be observed.

### 3.5. Brassinin Down-Regulates Key Angiogenic Signaling Pathways

**Figure 5:**
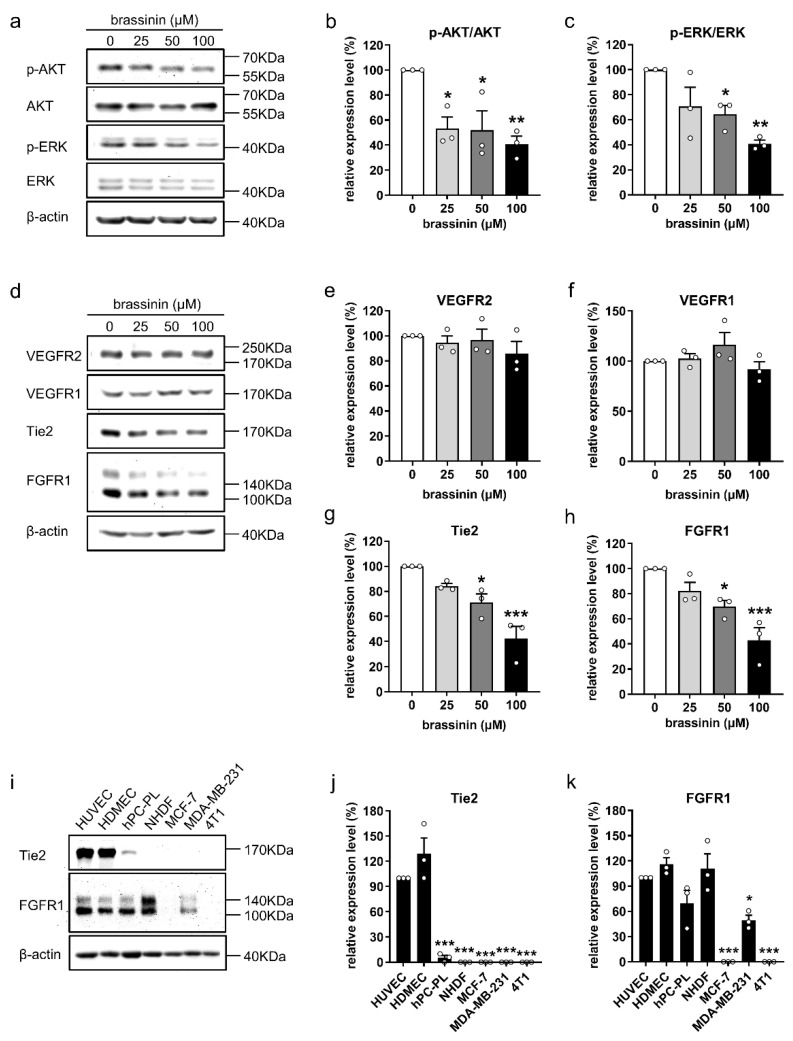
Brassinin down-regulates key angiogenic signaling pathways in ECs. (**a**) Representative Western blots of p-AKT, AKT, p-ERK, ERK, and β-actin expressed in HUVECs that were treated for 2 h with 0, 25, 50, and 100 µM brassinin. (**b**,**c**) Expression levels (% of 0 µM) of p-AKT/AKT (**b**) and p-ERK/ERK (**c**) in treated HUVECs were quantified (*n* = 3). (**d**) Representative Western blots of VEGFR2, VEGFR1, Tie2, FGFR1, and β-actin expressed in HUVECs that were treated for 2 h with 0, 25, 50, and 100 µM brassinin. (**e**–**h**) Expression levels (% of 0 µM) of VEGFR2 (**e**), VEGFR1 (**f**), Tie2 (**g**) or FGFR1 (**h**) in treated HUVECs were assessed (*n* = 3). (**i**) Representative Western blots of Tie2, FGFR1, and β-actin expressed in HUVECs, HDMECs, hPC-PLs, and NHDFs, as well as MCF-7, MDA-MB-231, and 4T1 cells. (**j**,**k**) Expression levels (% of HUVEC) of Tie2 (**j**) and FGFR1 (**k**) in different types of cells were determined (*n* = 3). Means ± SEM. * *p* < 0.05, ** *p* < 0.01, *** *p* < 0.001 vs. 0 µM or HUVEC.

To elucidate how brassinin inhibits EC angiogenesis, we first assessed the activity of the AKT and ERK signaling pathways in HUVECs exposed for 2 h to 0, 25, 50, or 100 µM brassinin. Western blot analyses revealed that brassinin significantly suppresses the phosphorylation of both AKT and ERK (Figure 5a–c and Figure S1). Of note, this inhibitory effect could already be observed after a short exposure time of 0.5 h (Figures S1 and S2). These findings indicate that brassinin possibly targets a common upstream regulator of AKT and ERK. Accordingly, we further analyzed the protein levels of several receptor tyrosine kinases that are critical for angiogenesis, including VEGFR2, VEGFR1, Tie2, and FGFR1, in vehicle- and brassinin-treated HUVECs. Interestingly, we found that brassinin selectively reduces the expression of Tie2 and FGFR1 (Figure 5d–h and Figure S1). 

To investigate whether brassinin selectively targets ECs through down-regulation of Tie2 or FGFR1, we compared the expression levels of Tie2 and FGFR1 in HUVECs, HDMECs, hPC-PLs, and NHDFs, as well as MCF-7, MDA-MB-231, and 4T1 cells. By means of Western blotting, Tie2 was found to be highly and specifically expressed in both HUVECs and HDMECs, slightly expressed in pericytes, but rarely expressed in fibroblasts and the three breast cancer cell lines (Figure 5i,j and Figure S1). In contrast, FGFR1 was comparably expressed in ECs, pericytes, and fibroblasts, but significantly lower expressed in breast cancer cells when compared to HUVECs (Figure 5i,k and Figure S1). 

### 3.6. Brassinin Promotes Tie2 and FGFR1 Degradation

**Figure 6:**
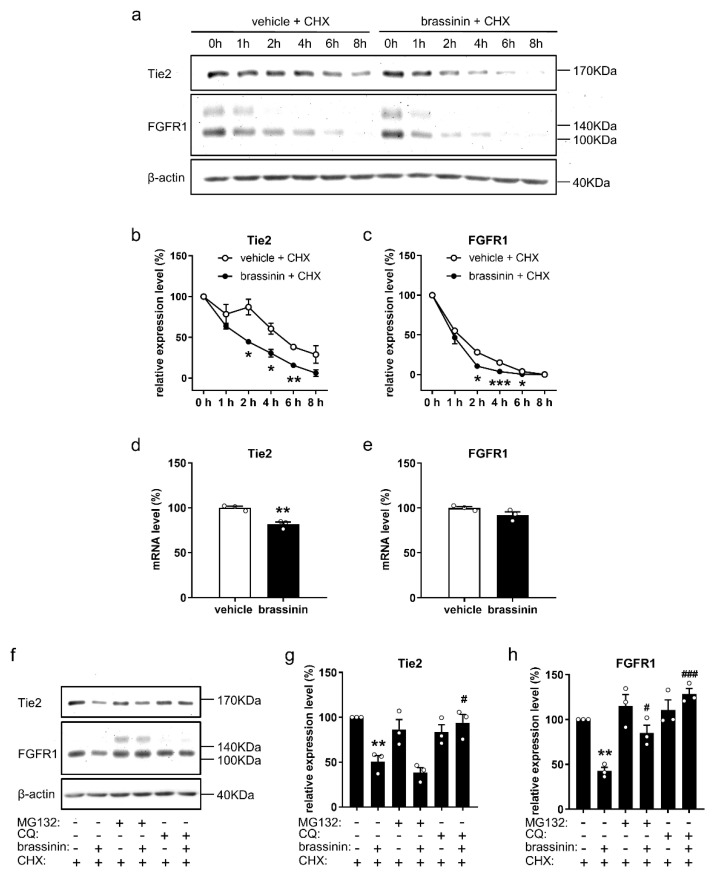
Brassinin promotes Tie2 and FGFR1 degradation in ECs. (**a**) Representative Western blots of Tie2, FGFR1, and β-actin expressed in HUVECs that were treated with 100 µM brassinin or DMSO (0.1% *v*/*v*; vehicle) in the presence of 100 µM CHX for the indicated time periods. (**b**,**c**) Expression levels (% of 0 h) of Tie2 (**b**) and FGFR1 (**c**) in treated HUVECs were quantified (*n* = 3). (**d**,**e**) mRNA levels (% of vehicle) of Tie2 (**d**) and FGFR1 (**e**) in HUVECs that were treated for 2 h with 100 µM brassinin or DMSO (0.1% *v/v*; vehicle) were assessed by quantitative real-time PCR (*n* = 3). (**f**) Representative Western blots of Tie2, FGFR1, and β-actin expressed in HUVECs that were pretreated without or with 20 µM MG132 or 200 µM CQ for 2 h and then exposed to 100 µM brassinin or DMSO (0.1% *v/v*; vehicle) in the presence of 100 µM CHX for another 2 h. (**g**,**h**) Expression levels (% of vehicle) of Tie2 (**g**) or FGFR1 (**h**) in treated HUVECs were quantified (*n* = 3). Means ± SEM. * *p* < 0.05, ** *p* < 0.01, *** *p* < 0.001 vs. vehicle or ‘vehicle + CHX’. # *p* < 0.05, ### *p* < 0.001 vs. ‘brassinin + CHX’.

To investigate how brassinin decreases the protein levels of Tie2 and FGFR1, we analyzed the effects of the compound on Tie2 and FGFR1 degradation in HUVECs, which were treated with vehicle or brassinin in the presence of the protein synthesis inhibitor cycloheximide (CHX). By means of Western blotting, we could demonstrate that the degradation of Tie2 and FGFR1 is significantly accelerated in brassinin-treated HUVECs when compared to vehicle-treated controls (Figure 6a–c and Figure S1). We additionally performed real-time PCR analyses of vehicle- and brassinin-treated HUVECs. Treatment with brassinin only slightly reduced the mRNA expression of Tie2 by 18% (Figure 6d). In contrast, brassinin caused no change in the mRNA level of FGFR1 in HUVECs (Figure 6e). 

In eukaryotic cells, the ubiquitin-proteasome and the autophagy-lysosome are mainly responsible for the degradation of proteins [23]. To investigate which system is exploited by brassinin for Tie2 and FGFR1 degradation, HUVECs were pretreated with the specific proteasome inhibitor MG132 and the lysosome inhibitor chloroquine (CQ), followed by brassinin treatment. Our results showed that MG132 significantly counteracts the degradation of FGFR1 in brassinin-treated ECs, while CQ completely reverses brassinin-induced Tie2 and FGFR1 degradation (Figure 6f–h and Figure S1). 

## 4. Discussion

Previous studies reported an anti-cancer activity of brassinin on several types of cancer, including colon [3], liver [4], prostate [24], and lung cancer [5,25]. However, the effects of brassinin on TNBC development have not been reported so far. TNBC is defined as tumors that do not exhibit estrogen and progesterone receptors as well as human epidermal growth factor receptor 2 [12]. Although TNBC only contributes to approximately 20% of new breast cancer cases, it has received much attention and interest due to its aggressive biological behavior, poor patient outcomes, and lack of effective treatments [12]. In the present study, we demonstrate for the first time that brassinin inhibits TNBC growth preferentially through inhibiting the angiogenic activity of ECs. Additional in-vitro analyses revealed that this effect may be mediated by brassinin-stimulated Tie2 and FGFR1 degradation in ECs.

In the first set of experiments, we analyzed in vitro the effects of brassinin on the viability of several important cell types of the tumor microenvironment, including breast cancer cells, ECs, pericytes, and fibroblasts. Pericytes are mural cells, which surround the ECs of blood vessels and are considered to remodel tumor vessels towards a mature phenotype. They have been reported to actively contribute to tumor metastasis and immune escape [26,27,28]. Fibroblasts are a predominant cell type within the tumor stroma. Given their pivotal contributions to tumor growth, angiogenesis, inflammation, and immunosuppression, they represent a promising target for cutting-edge therapeutic strategies against cancer [29,30]. Of interest, we found that both types of the herein analyzed ECs, i.e., HUVECs and HDMECs, react much more sensitive to brassinin than the other cell types. These results indicate that brassinin selectively targets ECs in the tumor microenvironment. However, this conclusion should be taken with some caution, because cancer-associated stroma cells may differ in their reaction to brassinin when compared to the herein used cell types from benign tissue sources. Additional in-vitro assays demonstrated pleiotropic inhibitory effects of non-cytotoxic doses of brassinin on the angiogenic activity of ECs, including proliferation, migration, tube formation, and spheroid sprouting. This anti-angiogenic activity of brassinin was further verified in an ex-vivo mouse aortic ring assay and an in-vivo Matrigel plug assay. Hence, it can be concluded that the inhibition of blood vessel formation is the major mechanism of the anti-cancer activity of brassinin.

To assess the action of brassinin on tumor development, we exploited a mouse dorsal skinfold chamber model of TNBC. This model enabled us to repeatedly monitor the growth and vascularization of newly developing tumors using intravital fluorescence microscopy [31]. For this purpose, spheroids of murine 4T1 mammary cancer cells were generated and transplanted into the dorsal skinfold chambers of vehicle- and brassinin-treated mice. Of note, the 4T1 cell line is derived from a spontaneously arising mammary tumor in Balb/c mice and is widely used for syngeneic murine TNBC models, which closely mimic human TNBC [32]. We could demonstrate that brassinin treatment markedly delays 4T1 tumor growth. In line with our in-vitro data, we further found a significantly reduced vascularization of brassinin-treated tumors. Additional immunohistochemical analyses showed that the percentage of proliferating tumor cells is decreased in brassinin-treated mice, whereas tumor cell apoptosis and necrosis are not affected. These results suggest that brassinin suppresses tumor growth mainly through its anti-angiogenic activity rather than through direct cytotoxic effects on tumor cells. These findings should be confirmed and expanded in future long-term in-vivo studies, because the herein used dorsal skinfold chamber model only allows the investigation of newly developing tumors over a limited time period of 14 days. Hence, we could not analyze whether brassinin also induces the regression of well-established larger tumors and whether chronic treatment with the compound induces side effects and tumor resistance. 

We additionally analyzed the molecular mechanisms of brassinin action. Western blot assays showed that a 2 h exposure to brassinin remarkably decreases the protein levels of Tie2 and FGFR1, but not VEGFR1 and VEGFR2 in ECs. This was associated with the inhibition of down-stream AKT and ERK signaling pathways. Tie2, a receptor tyrosine kinase predominantly enriched in the endothelium, is highly expressed in the vasculature of tumors [33]. Activation of Tie2 stimulates EC survival, sprouting, migration, and capillary tube formation [33]. On the other hand, the blockage of Tie2 action has been shown to inhibit tumor angiogenesis and growth in several mouse models of different cancer types, including breast cancer, melanoma, and hepatocellular carcinoma [34,35,36,37]. FGFR1, a member of the FGFR family, is also a well-known receptor tyrosine kinase primarily expressed on ECs [38]. It can be activated with FGF1 and bFGF, triggering the angiogenic activity of ECs in vitro and in vivo [38]. Accordingly, FGFR1 is considered as a promising target for the treatment of tumor angiogenesis. Of interest, it is further known that Tie2 and FGFR1 can physically interact with each other, which promotes the phosphorylation of signal transducer and activator of transcription 3 (STAT3) [39]. STAT3 signaling, in turn, is also crucially involved in the regulation of new blood vessels within different types of tumors [40,41]. Given the pivotal roles of both Tie2 and FGFR1 in tumor angiogenesis, we assume that the combined blockade of these two pathways may be the reason for the high anti-angiogenic efficiency of brassinin. This mode of brassinin action may also prevent the development of tumor resistance and improve the clinical outcomes of treated cancer patients in the future. 

We additionally compared the protein levels of Tie2 and FGFR1 in ECs, breast cancer cells, pericytes, and fibroblasts. Tie2 was initially characterized as an EC-specific receptor tyrosine kinase [42], whereas later on it was also reported to be expressed in pericytes [43] and different types of tumor cells [33]. Consistently, we found that Tie2 is highly expressed in ECs and slightly expressed in pericytes. In contrast, Tie2 expression was hardly detectable in fibroblasts as well as the three breast cancer cell lines, i.e., MDA-MB-231, MCF-7, and 4T1. Previous studies showed the expression of FGFR1 in ECs, pericytes, and fibroblasts [44,45]. Moreover, amplification of FGFR1 was found in approximately 10% of breast cancers [46]. However, the expression of FGFR1 in these cell types has not been compared so far. We herein demonstrate that FGFR1 is relatively highly expressed in ECs, pericytes, and fibroblasts when compared to breast cancer cells. Taken together, these findings indicate that brassinin may exert the above-described high selectivity against ECs via targeting Tie2 rather than FGFR1, although both Tie2 and FGFR1 down-regulation possibly contribute to the anti-angiogenic effects of the compound.

There are different intracellular processes involved in the regulation of protein levels, including the transcription, translation, and degradation of mRNA, as well as protein degradation. In the present study, we found that brassinin slightly reduces the mRNA expression of endothelial Tie2, but not of FGFR1. In contrast, the phytochemical compound markedly promoted the degradation of both receptors. This mode of brassinin action allows for the ability not only to reduce the number of existent Tie2 and FGFR1, but also to counteract compensatory overexpression of these proteins, which is often associated with the inhibition of protein function [47]. Accordingly, brassinin appears to be clearly superior to traditional small-molecule Tie2 or FGFR1 kinase inhibitors.

Although previous studies demonstrated that, similar to brassinin, angiopoietins also cause rapid degradation of Tie2 [48,49], the mechanism of Tie2 degradation has been less studied. In contrast, it is well known that the binding of FGFs to FGFR1 results in receptor dimerization, internalization, and subsequent degradation mainly in the lysosome [50]. Interestingly, we herein found that brassinin stimulates the lysosomal but not proteasomal degradation of Tie2. Moreover, it promoted both the lysosomal and proteasomal degradation of FGFR1. Of note, the proteasome and lysosome degradative systems share molecular determinants, such as ubiquitination, and substrates based on which they interact with each other and act as a network [23]. The degradation choice of each protein is self-organized and determined by biophysical parameters, including binding affinity, local concentration, and avidity, as well as compartmentalization through membranes, liquid-liquid phase separation, or aggregates formation [23]. Therefore, it is reasonable that the lysosome and proteasome are differentially involved in the brassinin-induced degradation of Tie2 and FGFR1.

## 5. Conclusions

**Figure 7:**
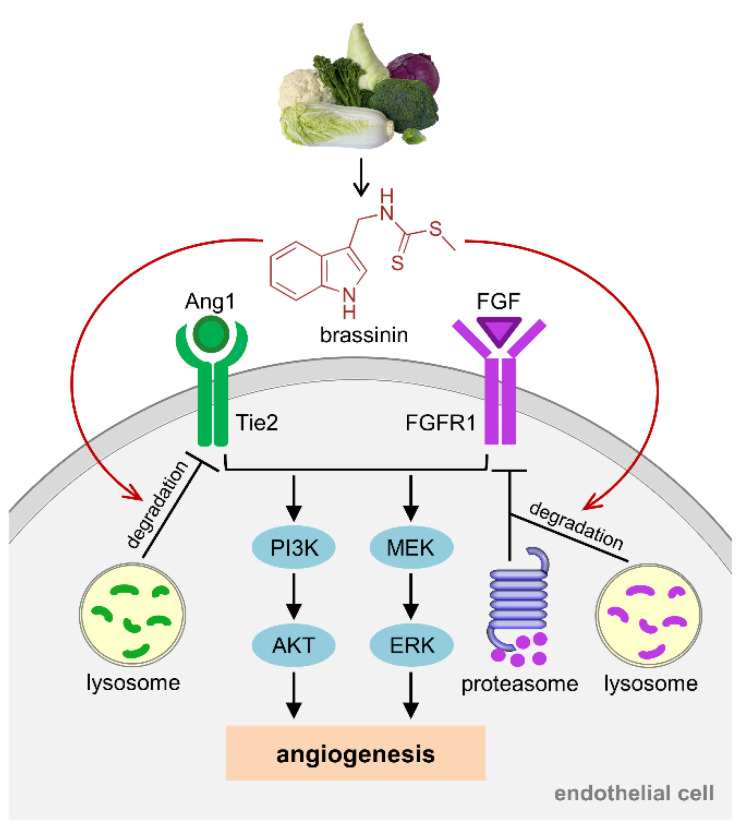
Schematic diagram representing the molecular mechanisms underlying the anti-angiogenic effects of brassinin. Brassinin stimulates the lysosomal degradation of Tie2 as well as the lysosomal and proteasomal degradation of FGFR1 in endothelial cells. This results in the down-regulation of the AKT and ERK pathway and, thus, the inhibition of angiogenesis.

Taken together, the present study demonstrates a potent anti-angiogenic activity of brassinin, which is based on its stimulatory action on Tie2 and FGFR1 degradation, resulting in the down-regulation of the AKT and ERK pathway (Figure 7). Brassinin acts as a pleiotropic anti-angiogenic compound, which targets all the key angiogenic activities of ECs. Accordingly, administration of this phytochemical compound efficiently inhibits TNBC vascularization and growth. Hence, brassinin represents a promising anti-angiogenic agent for the future treatment of cancer.

## Data Availability

All data generated or analyzed during this study are included in this published article.

## Supplementary Materials

The following supporting information can be downloaded at: https://www.mdpi.com/article/10.3390/cancers14143540/s1. Video S1: Perfusion of tumor vessels in a vehicle-treated mouse on day 14. Video S2: Perfusion of tumor vessels in a brassinin-treated mouse on day 14. Figure S1: Original images of Western blots. Figure S2: Brassinin inhibits AKT and ERK phosphorylation in ECs after short exposure.
