# Supplementary material for: Brassinin Promotes the Degradation of Tie2 and FGFR1 in Endothelial Cells and Inhibits Triple-Negative Breast Cancer Angiogenesis
Source: Cancers (Basel). 2022 Jul 21;14(14):3540. doi: 10.3390/cancers14143540 (PMC9318525; doi:10.3390/cancers14143540)
Supplement: Supplementary file 1 [file cancers-14-03540-s001.zip › Supplement/cancers-1798339-supplementary.pptx]

## Slide 1
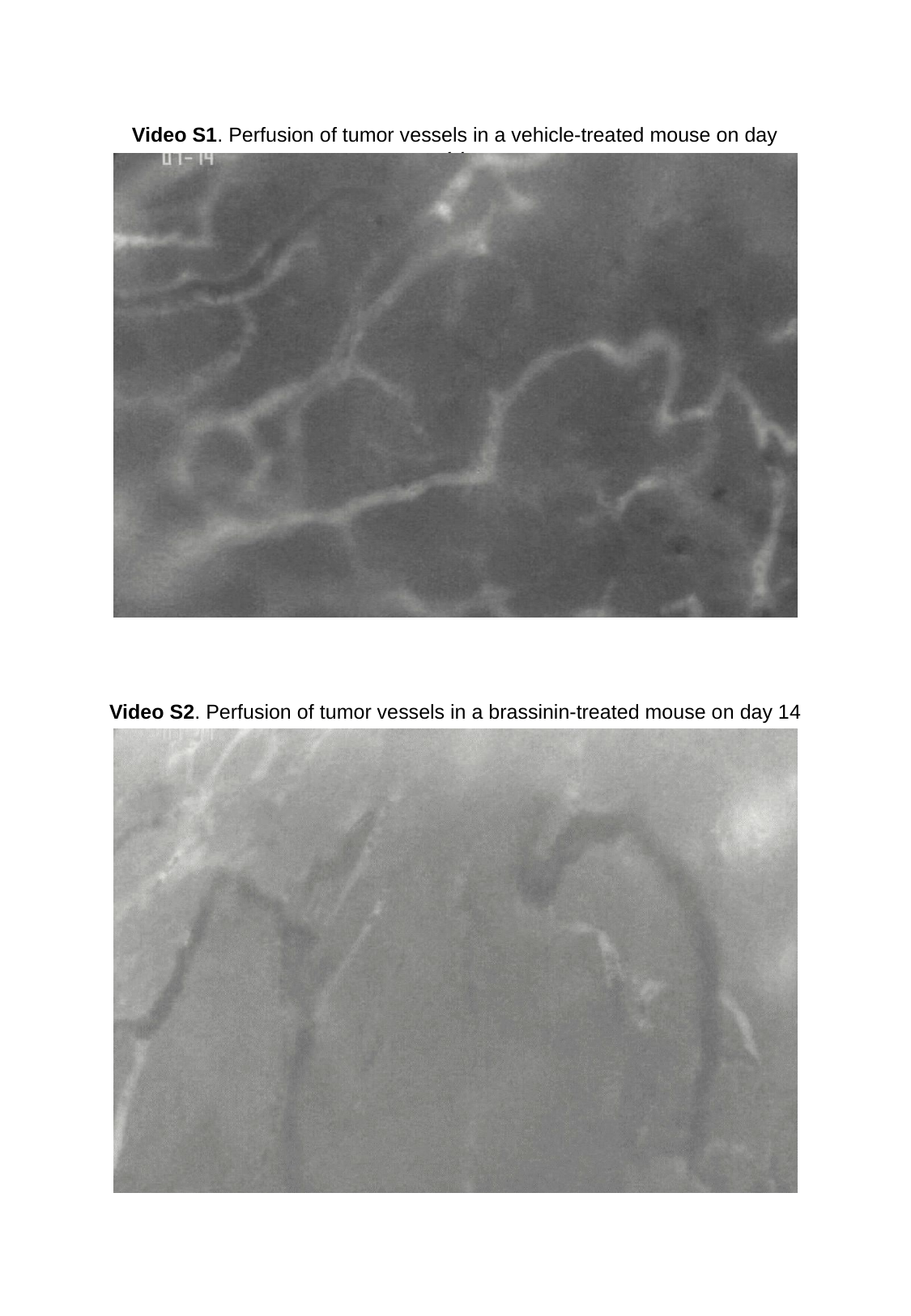

Video S1. Perfusion of tumor vessels in a vehicle-treated mouse on day 14
Video S2. Perfusion of tumor vessels in a brassinin-treated mouse on day 14
