# Supplementary material for: Brassinin Promotes the Degradation of Tie2 and FGFR1 in Endothelial Cells and Inhibits Triple-Negative Breast Cancer Angiogenesis
Source: Cancers (Basel). 2022 Jul 21;14(14):3540. doi: 10.3390/cancers14143540 (PMC9318525; doi:10.3390/cancers14143540)

Figure S1

original images of WB for Fig. 5a **original images of WB for Fig. 5d** **original images of WB for Fig. 5i**

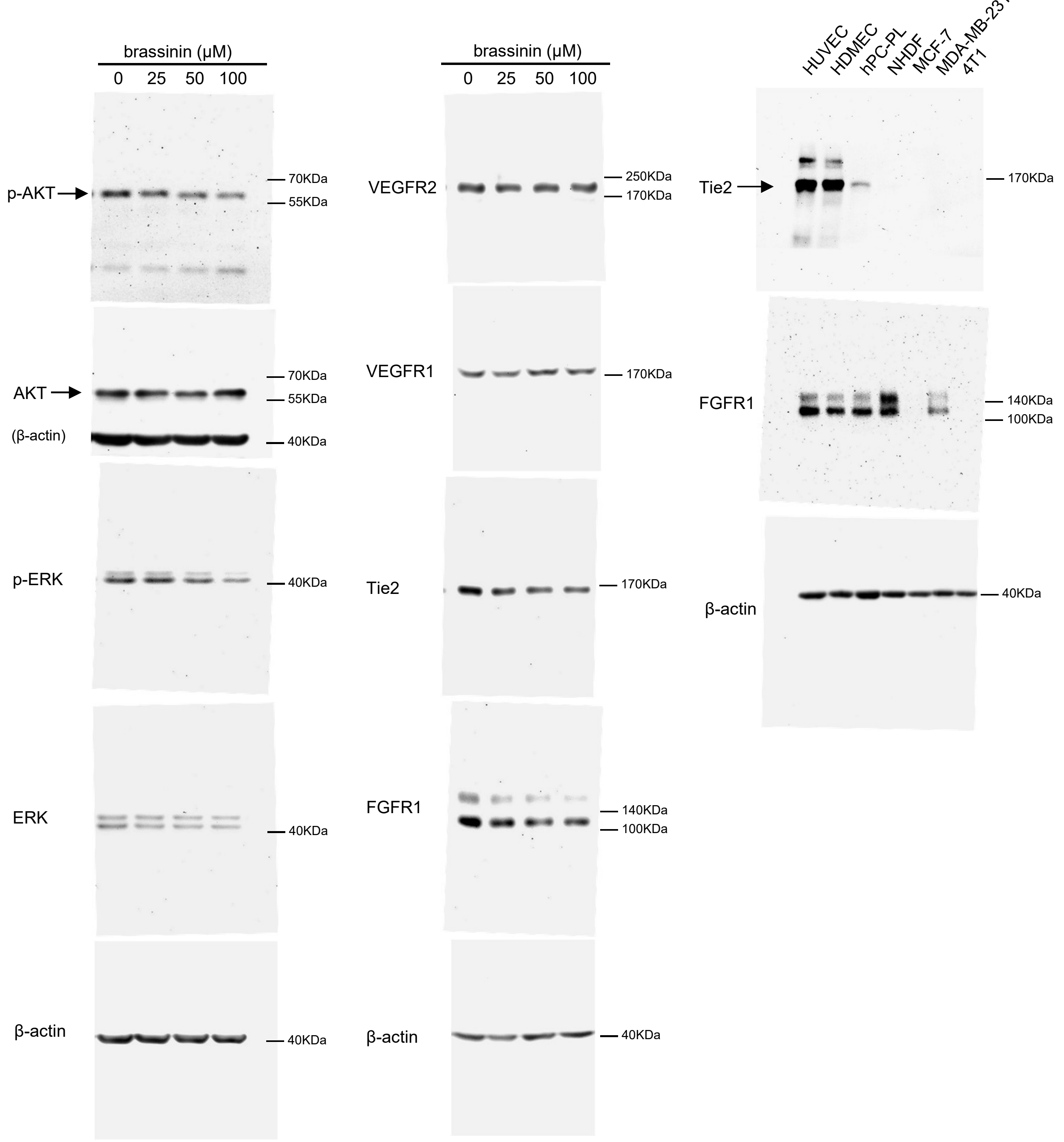

original images of WB for Fig. 6a

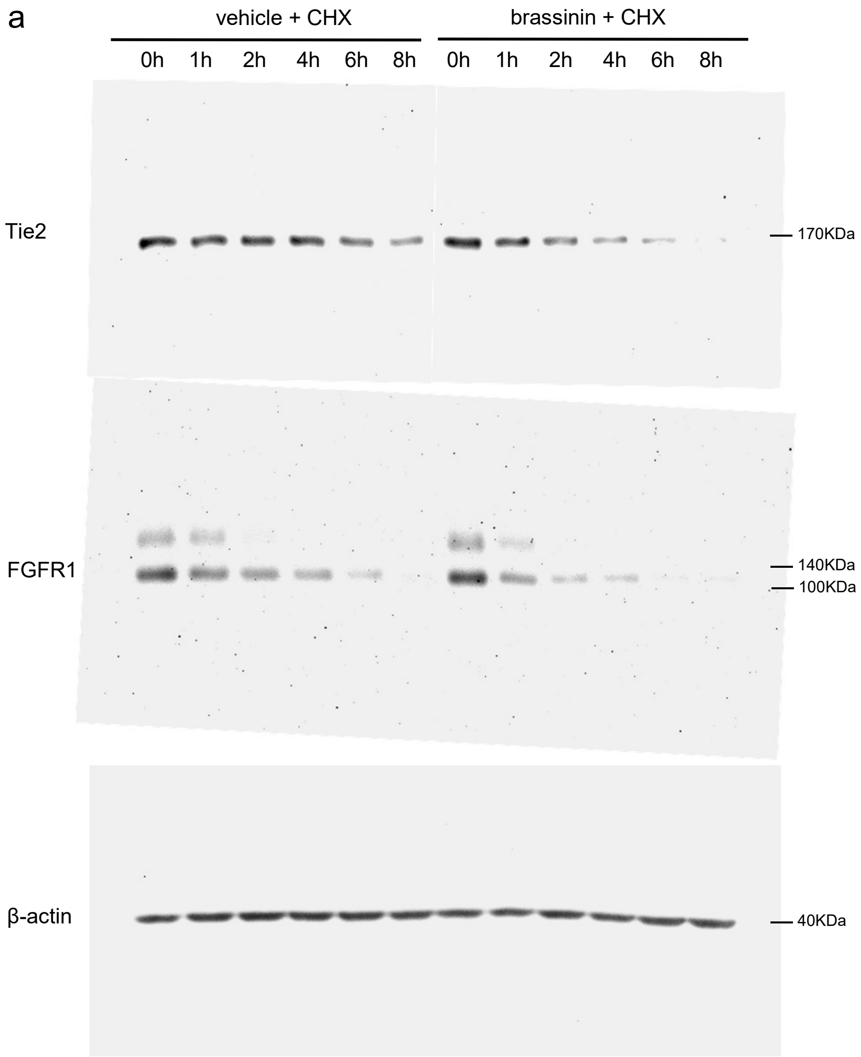

original images of WB for Fig. 6f

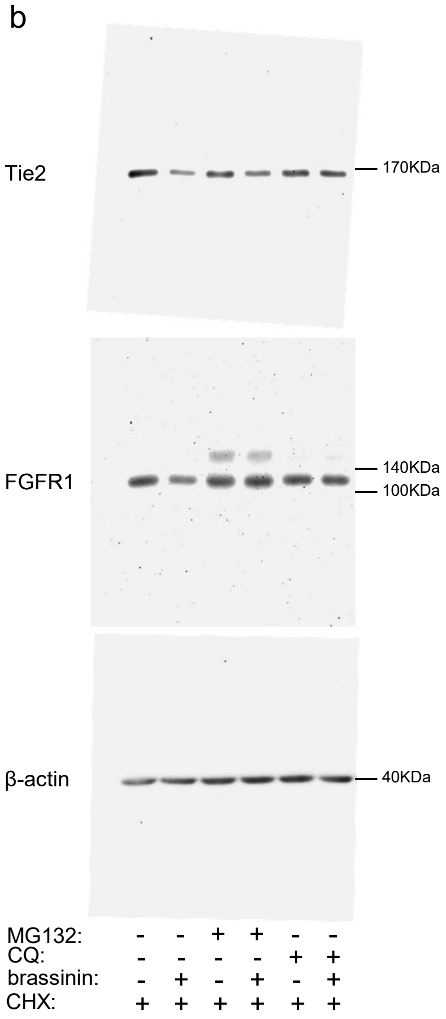

original images of WB for Fig. S1

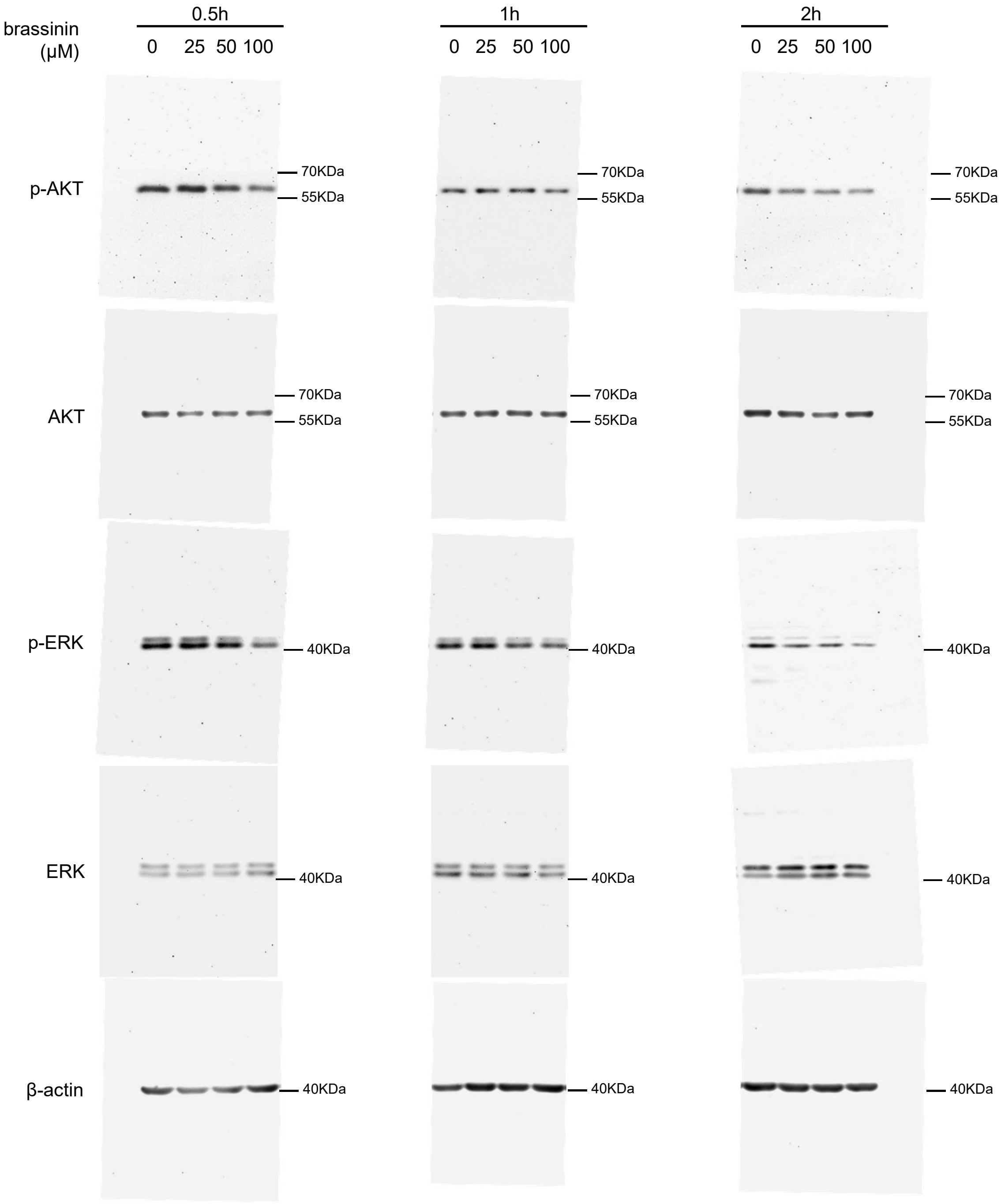

Supplement: Supplementary file 1 [file cancers-14-03540-s001.zip › Supplement/Figure S1.pdf]
