# Supplementary material for: Brassinin Promotes the Degradation of Tie2 and FGFR1 in Endothelial Cells and Inhibits Triple-Negative Breast Cancer Angiogenesis
Source: Cancers (Basel). 2022 Jul 21;14(14):3540. doi: 10.3390/cancers14143540 (PMC9318525; doi:10.3390/cancers14143540)
Supplement: Supplementary file 1 [file cancers-14-03540-s001.zip › Supplement/Figure S2.pdf]

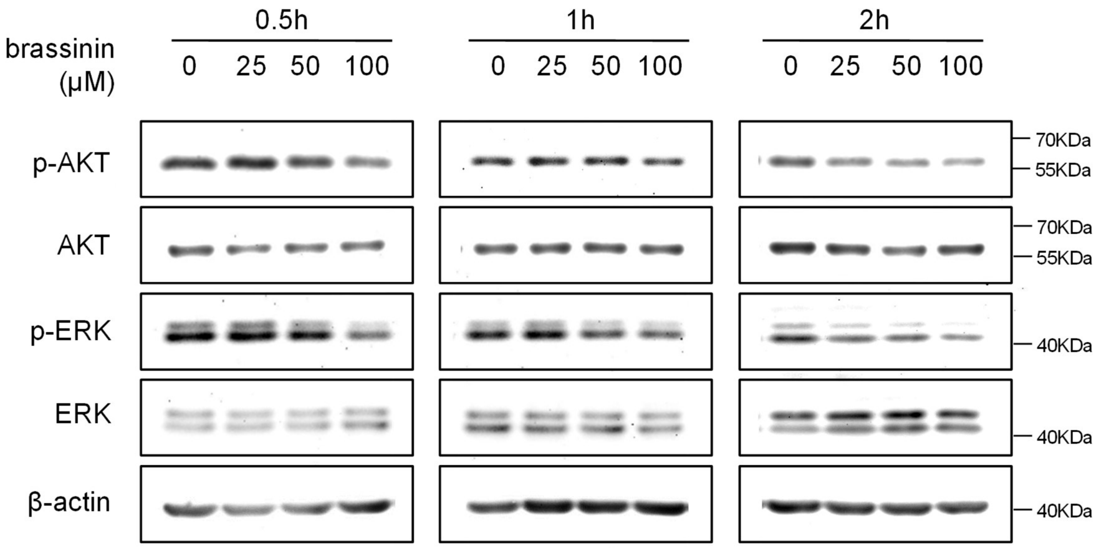

Figure S2. Brassinin inhibits AKT and ERK phosphorylation in ECs after short exposure. Western blots of p-AKT, AKT, p-ERK, ERK and β-actin expressed in HUVECs that were treated for 0.5, 1 and 2 h with 0, 25, 50 and 100 μM brassinin.
